# Application of the Internet Platform in Monitoring Chinese Public Attention to the Outbreak of COVID-19

**DOI:** 10.3389/fpubh.2021.755530

**Published:** 2022-01-28

**Authors:** Xue Gong, Mengchi Hou, Yangyang Han, Hailun Liang, Rui Guo

**Affiliations:** ^1^School of Public Health, Capital Medical University, Beijing, China; ^2^Department of Outpatient, Beijing Hospital of Traditional Chinese Medicine, Capital Medical University, Beijing, China; ^3^School of Public Administration and Policy, Renmin University of China, Beijing, China

**Keywords:** COVID-19, internet surveillance, Baidu Index, Sina Micro Index, epidemic monitoring

## Abstract

**Objectives:**

The internet data is an essential tool for reflecting public attention to hot issues. This study aimed to use the Baidu Index (BDI) and Sina Micro Index (SMI) to confirm correlation between COVID-19 case data and Chinese online data (public attention). This could verify the effect of online data on early warning of public health events, which will enable us to respond in a more timely and effective manner.

**Methods:**

Spearman correlation was used to check the consistency of BDI and SMI. Time lag cross-correlation analysis of BDI, SMI and six case-related indicators and multiple linear regression prediction were performed to explore the correlation between public concern and the actual epidemic.

**Results:**

The public's usage trend of the Baidu search engine and Sina Weibo was consistent during the COVID-19 outbreak. BDI, SMI and COVID-19 indicators had significant advance or lag effects, among which SMI and six indicators all had advance effects while BDI only had advance effects with new confirmed cases and new death cases. But compared with the SMI, the BDI was more closely related to the epidemic severity. Notably, the prediction model constructed by BDI and SMI can well fit new confirmed cases and new death cases.

**Conclusions:**

The confirmed associations between the public's attention to the outbreak of COVID and the trend of epidemic outbreaks implied valuable insights into effective mechanisms of crisis response. In response to public health emergencies, people can through the information recommendation functions of social media and search engines (such as Weibo hot search and Baidu homepage recommendation) to raise awareness of available disease prevention and treatment, health services, and policy change.

## Introduction

The outbreak of COVID-19 at the end of 2019 has triggered continual public attention in China. As of December 2020, Chinese internet users' numbers had reached 989 million (1). Nearly 80% of Internet users actively paid attention to COVID-19 epidemic information many times a day (2). Effective and efficient online monitoring is essential not only for the surveillance of public health events at the early stage but also for risk communication with the public.

Infodemiology is the science of distribution and determinants of information in an electronic medium, specifically the Internet, or in a population, with the ultimate aim to inform public health and public policy (3, 4). Examples for infodemiology applications include: the analysis of queries from Internet search engines to predict disease outbreaks; monitoring peoples' status updates on microblogs such as Twitter for syndromic surveillance; detecting and quantifying disparities in health information available, and so on (3, 4). Eysenbach (4) divided online data into two categories: demand-based data and supply-based data. Demand-based data is what people actively search on the Internet, while supply-based data is what is published on social media such as Facebook and Weibo. Previous research has shown that search engine query data and social media data can predict such epidemics as seasonal flu (5), human immunodeficiency virus (HIV) (6), Ebola (7), general influenza (8), and Zika virus (9). These studies mentioned above are more based on query data from Google, which are relevant in the context of English culture. However, the knowledge gap existed that needs to be filled to assess whether similar conclusions apply to other diseases, cultures, contexts, search engines, or social media.

Demand-based Search Engines in China are mainly Baidu, Sogou, 360, and so on, while in other countries, Google and Yahoo are most commonly used. In 2019, Baidu ranked first in penetration rate with 90.9 percent in China (10), equivalent to Google search in Western countries, which can better reflect the concerns of most Chinese people on the epidemic compared with other search engines. The BDI (Baidu Index) is the indicator to reflect the public use of the Baidu search engine to obtain information (11). Compared with the traditional survey, internet search behavior has higher authenticity, objectivity, timely and credibility, is an essential reference for social demand monitoring (3).

Recently, the Internet data obtained from Google Trends (3) and Baidu search (12) have been used to measure public attention in public health emergencies such as influenza, H7N9, and dengue fever. Eysenbach firstly used Google Trends to predict flu trends in 2009 (4), which attracted other scholars to investigate further. Several studies showed that there were positive correlations between search engine data and the number of confirmed cases of public health emergencies (such as influenza, dengue fever, ebola, etc.) (12–16). And the correlation is low when the outbreak begins; with cases gradually increasing, the correlation becomes high (17). In addition, Huang et al. (18) found that the keyword search volume of some cities was highly correlated with the actual incidence data, while some cities were not. COVID-19 is the most significant public health emergency in China after SARS in 2003, and further research is needed to determine whether there is a statistical correlation between case-related indicators and the BDI.

Supply-based Social Media includes Weibo, WeChat, Tik Tok, Toutiao, online forums, and so on. As the most popular social media platform in China, Sina Weibo has more than 800 million registered users as of March 2018, and more than 100 million messages are published every day (19), which cover all aspects of social news and hot topics and can be posted or searched anywhere and anytime (20). The SMI (Sina Micro Index) is the indicator to measure keywords in the spread of interactive effects on Weibo (21). Social media can provide a personalized and unique news experience, and people can interact directly with “news,” which is also plays an irreplaceable role in netizens' access to network information.

Many scholars used Sina Weibo to study the risk of network emergencies. Li et al. (22) took an empirical study using Sina Weibo data and found that in the initial stage of events, the government's control cost of risk communication is lower and more effective. Besides, social media data were also often used for disease prediction (23, 24). At present, there are relatively few studies on the public health crisis in the academic community using social media. A systematic review found that the published social media studies relating to the Ebola virus mainly focused on Twitter and YouTube, with only one article from Weibo (7). On the whole, few articles use Sina Weibo as the data source to study the correlation between public information searches and epidemic development, which is worthy of further research.

Current research is mostly based on a single platform. This paper uses two different types of platforms: demand-based search engines and supply-based social media. As representatives of current Chinese search engines and social networking platforms, their internet data are more valuable for monitoring China's first national public crisis since it entered the mobile social media era. Since COVID-19, researchers have used media reports (25), Google Trends (26), Weibo post counts, Baidu searches (27, 28), and Ali indices (29, 30) to study public attention and risk communication for COVID-19 epidemic management. But there has been no study of the Chinese public's response to COVID-19 using data such as BDI and SMI from the perspective of supply and demand. It can reveal the characteristic that netizen browses information more comprehensively and provide a reference for global outbreak information management.

Therefore, our specific research questions included the following:

(1) Is there any difference in public concern reflected between demand-based data and supply-based data during COVID-19?
(2) What are the correlations between BDI, SMI and COVID-19 epidemic indicators? Are there any significant advance or delay effects?
(3) Whether the internet data could predict the COVID-19 epidemic in the future?

## Materials and Methods

### Study Design and Data Source

To understand the public attention about COVID-19 in China, we conducted an epidemiological study based on the online data from January 2, 2020, to March 20, 2020. On January 20, 2020, at a press conference of the National Health Commission of the People's Republic of China, expert Nanshan Zhong confirmed COVID-19 “person-to-person,” and the National Health Commission of the People's Republic of China would release the number of new cases in each province daily. Considering the data reliability, we used the official data reported by the government, so we selected January 20 as the starting time of the study to collect case data. The reason for choosing March 20, 2020, as the end time is that Hubei Province, the hardest-hit area, reported zero new confirmed cases for 3 days in a row and that China announced it had assisted 82 countries and international organizations, which are preliminary indications that public attention may shift from China to the world. Besides, we have brought forward the starting date of the BDI and SMI data collection to January 2, 2020, to give a complete picture of the change of public interest. We chose 2 weeks earlier than for the molecular diagnosis data (the diagnosis criteria were set on January 16, 2020) because previous studies have shown that the data from Internet search engines and social media platforms were able to predict the disease outbreak 2 weeks earlier than the traditional surveillance systems (31).

At the beginning of the outbreak, there was no unified name for COVID-19. Comprehensively considering the BDI and SMI algorithm, the habit of the Chinese public, as well as the research during the outbreak (32), we selected “Novel coronavirus (新型冠状病毒),” “Pneumonia (肺炎),” “New pneumonia (新型肺炎),” “Novel Coronavirus Pneumonia (新型冠状病毒肺炎),” “Epidemic (疫情),” “Wuhan (武汉)” and “Wuhan Pneumonia (武汉肺炎),” seven words with large data values as the BDI related keywords. These keywords include pneumonia, Wuhan, virus, and other words that can represent epidemic events. The BDI search keywords require precise words, while SMI search words are fuzzy, such as “Wuhan Pneumonia.” The BDI data is large, and the SMI approaches zero. Therefore, when selecting SMI keywords, use “Pneumonia” instead of “New pneumonia,” “Novel Coronavirus Pneumonia,” “Wuhan Pneumonia,” and “Virus” instead of “Novel Coronavirus.” Therefore, we selected “Pneumonia (肺炎),” “Epidemic (疫情),” “Virus (病毒)” and “Wuhan (武汉)” as the keywords for the SMI.

The data for the BDI and SMI is from the Baidu Index (11) and the Sina Weibo Index (21) official website, respectively. COVID-19 case-related indicator data comes from the daily outbreak notification of the official website of the National Health Commission of the People's Republic of China (33).

### Statistical Analysis

We graphed the curves of the COVID-19 outbreak to describe the severity of epidemics. Spearman correlation was used to check the consistency of BDI and SMI. Time lag cross-correlation analysis of BDI, SMI and six indicators was performed to explore the correlation between public concern and the actual epidemic. Furthermore, multiple linear regression prediction models were used to build the prediction model of BDI and SMI for COVID-19, and leave-one-out cross validation (LOOCV) (34) was performed to check the prediction performance of the model.

Considering the data comparability, except for the BDI and SMI bivariate correlation analysis time from January 2 to March 20, the other correlation analysis time is from January 20 to March 20. SPSS 26.0 software was used for analysis.

## Results

### Public Concern About the COVID-19 in China

**Figure 1:**
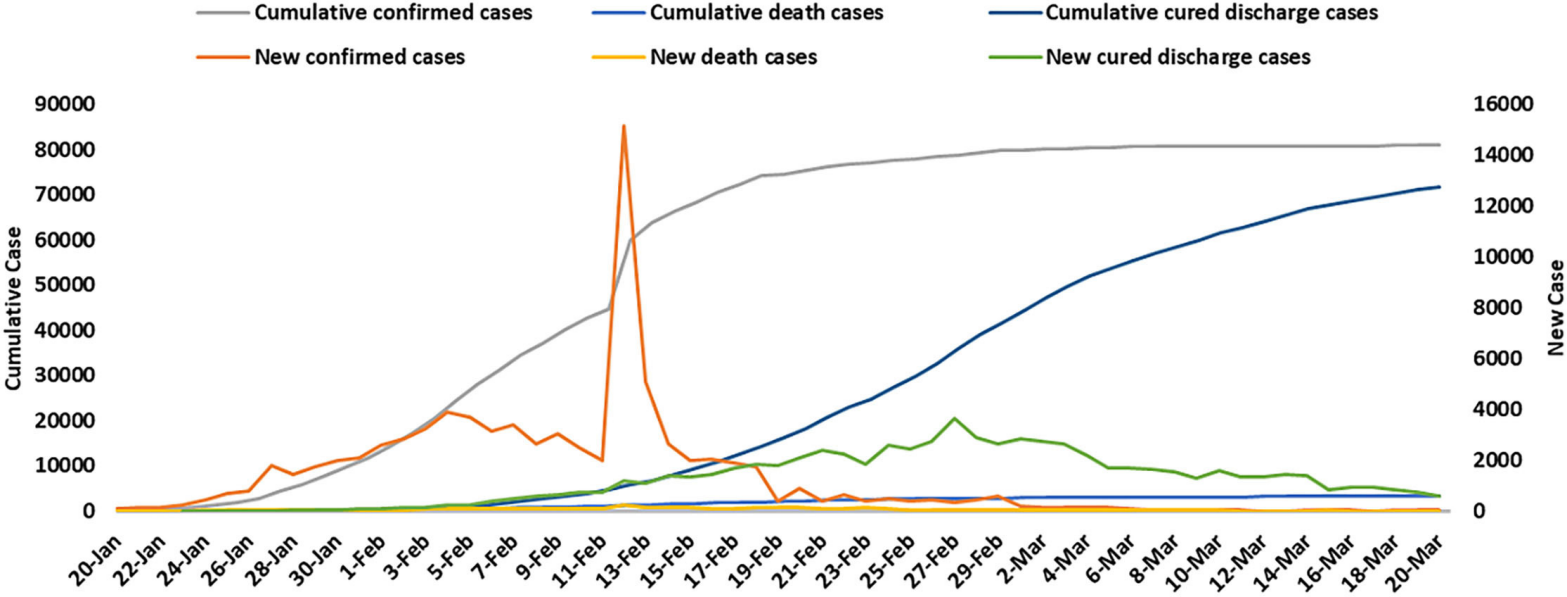
The epidemic characteristics of COVID-19 in China from January 20 to March 20 in 2020.

The World Health Organization (WHO) listed the COVID-19 outbreak as a public health emergency of international concern on January 30. On March 11, the WHO declared COVID-19 a global pandemic (35). Figure 1 depicts the characteristics of the COVID-19 outbreak in China between January 20, 2020 and March 20, 2020, through new confirmed cases, new death cases, new cured discharge cases, cumulative confirmed cases, cumulative death cases, and cumulative cured discharge cases (36).

**Figure 2:**
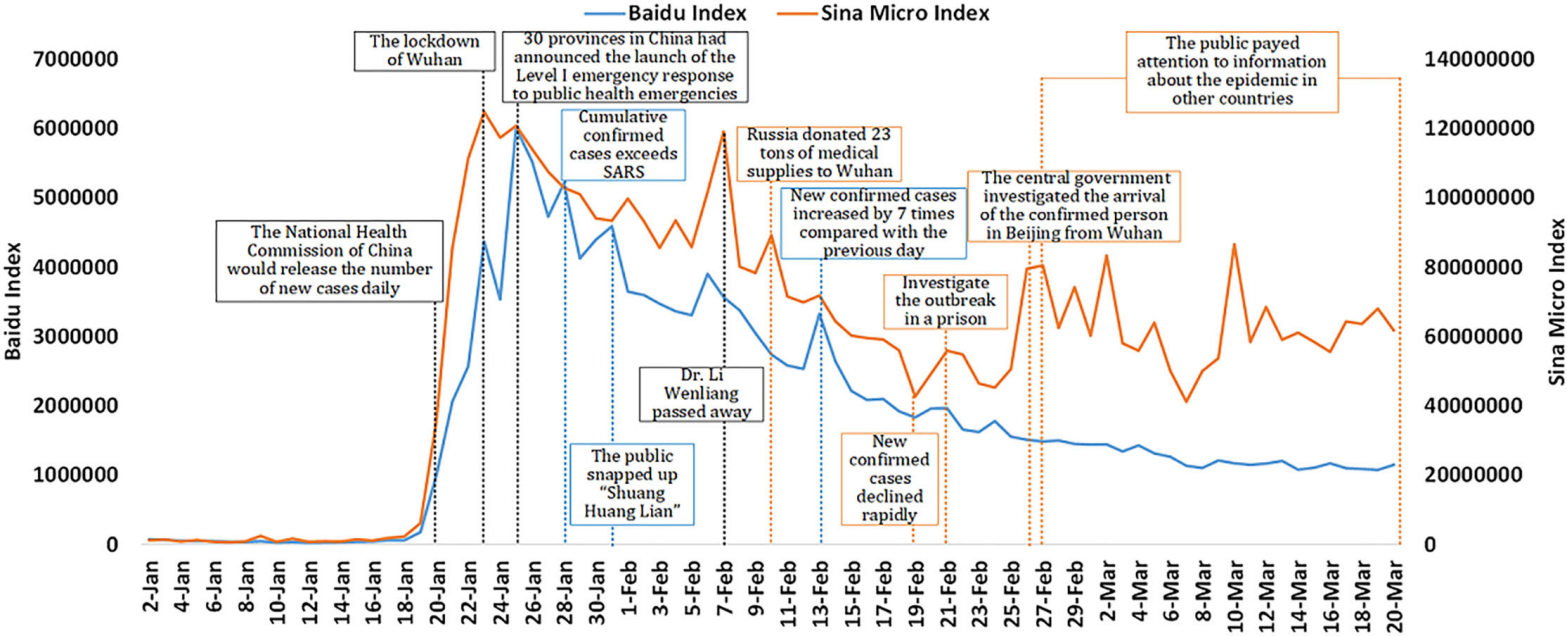
The changing trend of the Baidu Index and Sina Micro Index of COVID-19 in China from January 2 to March 20 in 2020. Blue explains the Baidu Index, orange explains the Sina Weibo Index, and black explains both.

We used BDI and SMI to describe the public concern about the COVID-19 outbreak in China. Figure 2 showed a positive correlation of BDI and SMI (Spearson correlation coefficient = 0.703, *P* < 0.001). In 2020, The BDI and SMI of “COVID-19” related keywords experienced a state of “developing from nothing, reaching a peak, fulling volatility, and stabilizing gradually.” The BDI and SMI were at a low level at the beginning of the observation period, and fluctuated significantly from January 20 to February 20, possibly because the pandemic was not well-controlled, the public had more panic, and the rapid development of the epidemic caused strong public attention. On January 20, the State Council included COVID-19 in the Infectious Diseases Law, and the National Health Commission of the People's Republic of China immediately released the number of new cases in each province, which aroused widespread public concern. Subsequently, the BDI and SMI increased significantly. On January 23, BDI reached its first small peak, and SMI reached its peak, which may be due to the public panic caused by the lockdown of Wuhan. The BDI reached its peak on January 25, in which 30 provinces in China had announced the launch of the Level I emergency response to public health emergencies. Subsequently, the BDI and SMI declined with fluctuation, and there were several small peaks. Possible reasons for the small peaks are indicated in Figure 2. Later, with the decrease of new confirmed cases and the increase of new cured discharge cases, the BDI showed a steady decline, while the SMI fluctuated, which may be related to the continuous hot topics on Sina Weibo. On March 20, the BDI and SMI were still higher than on January 20 (Figure 2). Overall, it can be considered that the demand-based search engine data and supply-based social media data showed the same public concern.

**Figure 3:**
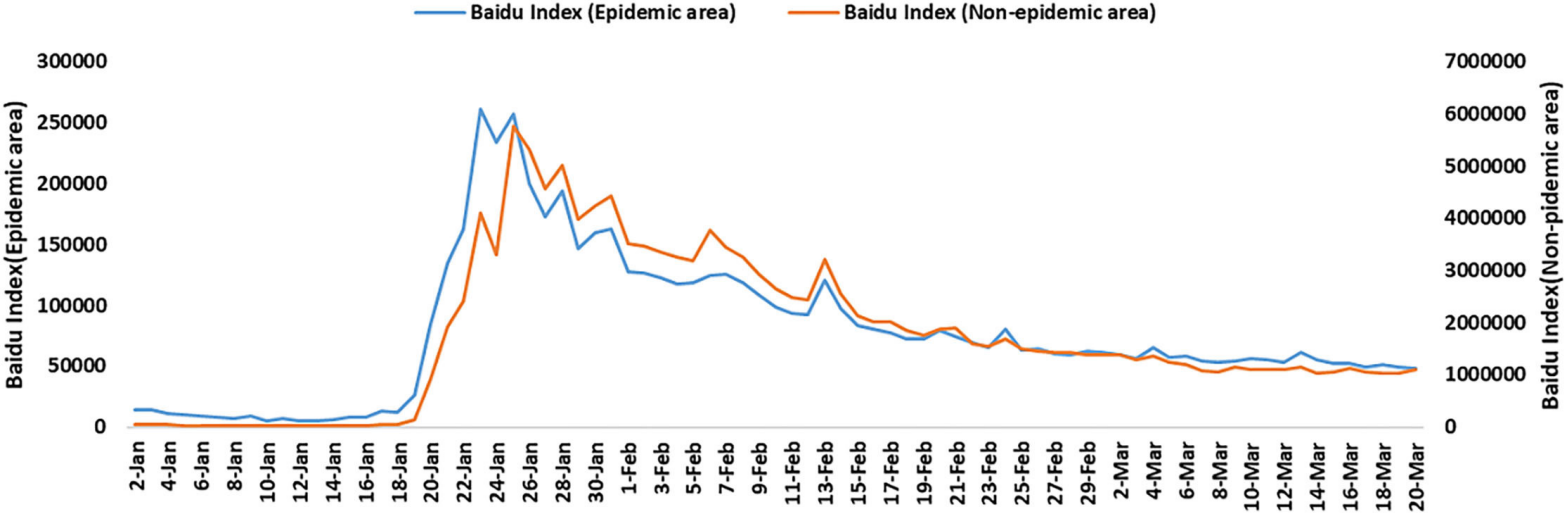
The changing trend of the National Baidu Index and Hubei Baidu Index of COVID-19 in China from January 20 to March 20 in 2020.

To eliminate the influence of difference between people in epidemic and non-epidemic areas, we compared the level of public network concern in Hubei province (epidemic area) with all other provinces except Hubei (non-epidemic area) in the same period (Figure 3). Correlation analysis results showed a positive correlation between Hubei and all other provinces (Spearman correlation coefficient = 0.930, *P* < 0.001) (Considering that SMI cannot be divided by different regions, and it has been proved above that there is no significant difference in public concern between BDI and SMI during the COVID-19, thus only BDI was used for analysis).

### Correlation Analysis Between Case-Related Indicators and Public Concern

We analyzed the time lag cross-correlation between the BDI, SMI and six case-related indicators within the time range of 20 days earlier or lagging to explore the possible indicators that cause fluctuations in public attention (Appendix 1 showed the specific correlation coefficients). The results (Table 1) showed that for new confirmed cases and new death cases, the highest correlation was found 6 and 16 days earlier in BDI, but the correlation with the other four indicators was highest at a lag of 3–16 days. While there was an advance effect before the change of SMI and six case indicators, and the correlation coefficients reached the highest 19, 19, 2, 11, 11, and 11 days earlier, respectively. In addition, BDI was more strongly correlated with case indicators than SMI.

**Table 1:** The time lag cross-correlation between the BDI, SMI and COVID-19-related data from 20 January to 20 March in 2020.

		**New confirmed cases**	**New death cases**	**New cured discharge cases**	**Cumulative confirmed cases**	**Cumulative death cases**	**Cumulative cured discharge cases**
BDI	Day	Lag-6	Lag-15	Lag16	Lag5	Lag5	Lag3
	Spearman correlation coefficient	0.900	0.879	−0.973	−0.986	−0.986	−0.986
	*P*-value	<0.001	<0.001	<0.001	<0.001	<0.001	<0.001
SMI	Day	Lag-19	Lag-19	Lag-2	Lag-11	Lag-11	Lag-11
	Spearman correlation coefficient	0.753	0.739	−0.722	−0.707	−0.707	−0.707
	*P*-value	<0.001	<0.001	<0.001	<0.001	<0.001	<0.001

### Epidemic Prediction Using Public Attention

From the above, only for new confirmed cases and new death cases, both BDI and SMI had advance effects. So we established the following four multiple linear regression models using new confirmed cases and new death cases as dependent variables, and seven BDI-related keywords or four SMI-related keywords as independent variables. Stepwise regression was used to remove variables that had no significant effect on the dependent variable (*P* > 0.1) and were closely related to other independent variables.

Model 1 (Table 2): seven BDI-related keywords were used to predict new confirmed cases.

**Table 2:** Forecast results of model 1 and model 2.

	**Model 1**	**Model 2**
(Constant)	−3,927.923^***^	−30.881^***^
Novel coronavirus		
Pneumonia		
New pneumonia		
Novel coronavirus pneumonia		
Epidemic	0.007^***^	1.26*10^−4^^***^
Wuhan	0.012^***^	
Wuhan pneumonia	0.021^***^	
*R* ^2^	0.443	0.315
Adj. *R*^2^	0.413	0.303
S.E	1,686.423	41.242
*F*	15.094	27.103
*P*-value	<0.001	<0.001

*** *Significance level of 1%*.

Model 2 (Table 2): seven BDI-related keywords were used to predict new death cases.

Model 3 (Table 3): four SMI-related keywords were used to predict new confirmed cases.

**Table 3:** Forecast results of model 3 and model 4.

	**Model 3**	**Model 4**
(Constant)	1,072.5056^***^	80.034^***^
Pneumonia	−2.33*10^−4^^***^	3.318*10^−6^^***^
Epidemic		
Virus		−5.472*10^−6^^***^
Wuhan	2.31*10^−4^^***^	6.020*10^−6^^***^
*R* ^2^	0.344	0.522
Adj. *R*^2^	0.322	0.497
S.E	1,813.456	35.044
*F*	15.228	20.750
*P*-value	<0.001	<0.001

*** *Significance level of 1%*.

Model 4 (Table 3): four SMI-related keywords were used to predict new death cases.

LOOCV was used to evaluate the fitting effect of the models, and it was found that Spearman correlation coefficients between predicted values (Appendix 2) and actual values of the four models were 0.892, 0.762, 0.766, and 0.674, respectively. It shows that the above four models perform well in predicting COVID-19 new cases.

## Discussions

### Main Findings

Using publicly accessible BDI, SMI, and COVID-19 case-related indicator data, this study analyzed the correlation between BDI, SMI and COVID-19 epidemic indicators and built prediction models of BDI and SMI for COVID-19. We found the public across the country had paid equal attention to demand-based and supply-based COVID-19 information. BDI, SMI and COVID-19 indicators had significant advance or lag effects, among which SMI and six indicators all had advance effects while BDI only had advance effect with new confirmed cases and new death cases. Notably, the prediction model constructed by BDI and SMI can well fit new confirmed cases and new death cases. Our study demonstrated that BDI and SMI, as effective early indicators, have been proved to monitor COVID-19 epidemics in China.

### Interpretations and Policy Recommendations

The BDI and SMI had a significant positive correlation, consistent with the findings of Kui et al. (37). The behaviors and concerns of the public during public health emergencies are consistent, whether actively seeking information or passively obtaining information. However, by the end of the observation period, SMI still had obvious fluctuations while BDI was relatively stable. Causes were attributed to that the epidemic has been well controlled, the public has more information about COVID-19, and thus the behavior of searching in Baidu search engine for the epidemic stabilized (38); a large amount of instant news provided by Sina Weibo will affect the public's information reception, and thus stimulating the public's information search behavior. The results of this study contribute to providing policy implications for government to respond to public opinion on public health emergencies by effectively using the information dissemination characteristics of search engines and social media. During public health emergencies, the Baidu search engine can give priority to provide users with the most attention and the most reliable sources of information, rather than the latest information; Sina Weibo can adjust the ranking of hot searches in the critical period, thereby affecting the public's information search behavior.

Another interesting finding was that SMI had an earlier effect than BDI in time lag cross-correlation analysis, which is a unique finding of this study. Compared with traditional websites, Sina Weibo, as a social media similar to Facebook and Twitter, is a typical “speed-type” media that spreads information faster, covers more widely (39), and is a collection and distribution center of “gossip.” By contrast, BDI is demand-based data, and its increase implies the tremendous need for COVID-19-related knowledge to the public, which usually occurs after the progress of the epidemic. Significantly, both BDI and SMI had advance effects for new confirmed cases and new death cases, meaning the public's great concern. It reminds us that the government should pay more attention to the information disclosure and interpretation of new confirmed cases and new death cases when responding to public health emergencies.

This study also showed that BDI had a higher correlation with the case indicators compared with SMI, consistent with previous studies about Google trends (40–42). But unfortunately, the previous study (43) only reported the correlation between BDI, SMI and the number of influenza patients. In this research, BDI, SMI and six important disease indicators were, respectively, used for correlation analysis, which was more specific and convincing. According to the 46th China Internet Development Statistics Report, Baidu searches (81.5%) have a higher netizen usage rate than Sina Weibo (42.5%) (44), which is more representative of the search intention of the wider public. Besides, Sina Weibo, which is easily driven by hot events, may cause huge fluctuations of the SMI in a short period (32).

Relying on traditional laboratory and clinical data to publish weekly statistics for countries and regions usually results in a lag of 1–2 weeks (27). But our data showed that the popularly used Internet search engines, Baidu, and the social media platform, Sina Weibo, were able to predict the disease outbreak 6–19 days earlier than the traditional surveillance systems. Big data monitoring of the epidemic can track the trends of infectious diseases and epidemics faster than traditional monitoring systems (27), which could buy time for controlling outbreaks of these diseases and reducing the risk of transmission to humans (37). This finding suggests that the government could build a tool for infectious disease surveillance based on BDI and SMI, which should be considered as supplementary to the traditional public health monitoring systems. When an outbreak occurs in the future, the government can make preparations for risk communication through this tool in advance. In the early stage of the epidemic, the communication content should focus on the disclosure and explanation of case-related indicators, especially new confirmed cases and new death cases, to effectively reduce public panic. Previous public health experience indicated that no individual country could single-handedly prevent and protect itself from public health threats (37). Accordingly, countries could jointly build a monitoring platform based on Internet data for information sharing to achieve early warning and risk communication of public health emergencies in advance.

## Limitations

Our research only focused on Sina Weibo and Baidu search engines and cannot describe netizens' attention to other platforms, such as WeChat.

## Conclusions

In this study, we found that the public concern presented by demand-based network data and supply-based network data is consistent. Supply-based network data and six indicators all had advance effects, while demand-based network data only had advance effects with new confirmed cases and new death cases. The confirmed associations between the public's attention to the outbreak of COVID on social media and the trend of epidemic outbreak implied valuable insights of effective mechanisms of crisis response and can be used to gain judgment related to people's information needs and to target groups who need additional attention. The information exchanges of netizens on the two platforms completely exposed their preferences and behaviors for epidemic information in online data. Interaction through online platforms potentially results in the concentration of prevailing concerns about the pandemic. In response to public health emergencies, people can through the information recommendation functions of social media and search engines (such as Weibo hot search and Baidu homepage recommendation) to achieve accurate information push. Social media about infectious disease raises awareness of available disease prevention and treatment and sensitizes the public to their need for health care. Moreover, this can lead to increased demand for health services which may, in turn, lead to changes in healthcare provision and policy change.

## Data Availability Statement

Publicly available datasets were analyzed in this study. This data can be found here: https://index.baidu.com/v2/index.html#/; https://data.weibo.com/index/; http://www.nhc.gov.cn/xcs/yqtb/list_gzbd.shtml.

## Author Contributions

MH and XG performed data analysis and edited the manuscript. All authors designed the research, critically revised the manuscript, reviewed and contributed to the final version, and approved it.

## Funding

This research was funded by National Natural Science Foundation of China (Grant Nos. 71704118 and 72174131).

## Conflict of Interest

The authors declare that the research was conducted in the absence of any commercial or financial relationships that could be construed as a potential conflict of interest.

## Publisher's Note

All claims expressed in this article are solely those of the authors and do not necessarily represent those of their affiliated organizations, or those of the publisher, the editors and the reviewers. Any product that may be evaluated in this article, or claim that may be made by its manufacturer, is not guaranteed or endorsed by the publisher.

## Abbreviations

BDI: Baidu Index
SMI: Sina Micro Index
WHO: World Health Organization.
