# Supplementary material for: Application of the Internet Platform in Monitoring Chinese Public Attention to the Outbreak of COVID-19
Source: Front Public Health. 2022 Jan 28;9:755530. doi: 10.3389/fpubh.2021.755530 (PMC8831856; doi:10.3389/fpubh.2021.755530)
Supplement: Supplementary file 1 [file Data_Sheet_1.DOCX]

Appendix 1

Table 1 The time lag cross-correlation between the BDI and COVID-19-related data from 20 January to 20 March in 2020.

| Baidu Index | | New confirmed cases | New death cases | New cured discharge cases | Cumulative confirmed cases | Cumulative death cases | Cumulative cured discharge cases |
| --- | --- | --- | --- | --- | --- | --- | --- |
| **Lag –20 days** | | | | | | | |
|  | Spearman correlation coefficient | 0.690 | 0.813 | 0.541 | -0.717 | -0.717 | -0.717 |
|  | *P* value | ＜0.001 | ＜0.001 | ＜0.001 | ＜0.001 | ＜0.001 | ＜0.001 |
| **Lag –19 days** | | | | | | | |
|  | Spearman correlation coefficient | 0.697 | 0.830 | 0.455 | -0.730 | -0.730 | -0.730 |
|  | *P* value | ＜0.001 | ＜0.001 | 0.002 | ＜0.001 | ＜0.001 | ＜0.001 |
| **Lag –18 days** | | | | | | | |
|  | Spearman correlation coefficient | 0.720 | 0.865 | 0.380 | -0.742 | -0.742 | -0.742 |
|  | *P* value | ＜0.001 | ＜0.001 | 0.012 | ＜0.001 | ＜0.001 | ＜0.001 |
| **Lag –17 days** | | | | | | | |
|  | Spearman correlation coefficient | 0.720 | 0.865 | 0.380 | -0.742 | -0.742 | -0.742 |
|  | *P* value | ＜0.001 | ＜0.001 | 0.012 | ＜0.001 | ＜0.001 | ＜0.001 |
| **Lag –16 days** | | | | | | | |
|  | Spearman correlation coefficient | 0.749 | 0.870 | 0.200 | -0.763 | -0.763 | -0.763 |
|  | *P* value | ＜0.001 | ＜0.001 | 0.187 | ＜0.001 | ＜0.001 | ＜0.001 |
| **Lag –15 days** | | | | | | | |
|  | Spearman correlation coefficient | 0.753 | **0.879** | 0.135 | -0.773 | -0.773 | -0.773 |
|  | *P* value | ＜0.001 | ＜0.001 | 0.372 | ＜0.001 | ＜0.001 | ＜0.001 |
| **Lag –14 days** | | | | | | | |
|  | Spearman correlation coefficient | 0.764 | 0.867 | 0.068 | -0.781 | -0.781 | -0.781 |
|  | *P* value | ＜0.001 | ＜0.001 | 0.650 | ＜0.001 | ＜0.001 | ＜0.001 |
| **Lag –13 days** | | | | | | | |
|  | Spearman correlation coefficient | 0.790 | 0.857 | ＜0.001 | -0.790 | -0.790 | -0.790 |
|  | *P* value | ＜0.001 | ＜0.001 | 0.999 | ＜0.001 | ＜0.001 | ＜0.001 |
| **Lag –12 days** | | | | | | | |
|  | Spearman correlation coefficient | 0.822 | 0.849 | -0.056 | -0.797 | -0.797 | -0.797 |
|  | *P* value | ＜0.001 | ＜0.001 | 0.702 | ＜0.001 | ＜0.001 | ＜0.001 |
| **Lag –11 days** | | | | | | | |
|  | Spearman correlation coefficient | 0.838 | 0.832 | -0.109 | -0.804 | -0.804 | -0.804 |
|  | *P* value | ＜0.001 | ＜0.001 | 0.453 | ＜0.001 | ＜0.001 | ＜0.001 |
| **Lag –10 days** | | | | | | | |
|  | Spearman correlation coefficient | 0.870 | 0.827 | -0.166 | -0.811 | -0.811 | -0.811 |
|  | *P* value | ＜0.001 | ＜0.001 | 0.244 | ＜0.001 | ＜0.001 | ＜0.001 |
| **Lag –9 days** | | | | | | | |
|  | Spearman correlation coefficient | 0.891 | 0.805 | -0.215 | -0.816 | -0.816 | -0.816 |
|  | *P* value | ＜0.001 | ＜0.001 | 0.126 | ＜0.001 | ＜0.001 | ＜0.001 |
| **Lag –8 days** | | | | | | | |
|  | Spearman correlation coefficient | 0.897 | 0.806 | -0.255 | -0.822 | -0.822 | -0.822 |
|  | *P* value | ＜0.001 | ＜0.001 | 0.066 | ＜0.001 | ＜0.001 | ＜0.001 |
| **Lag –7 days** | | | | | | | |
|  | Spearman correlation coefficient | 0.898 | 0.776 | -0.296 | -0.826 | -0.826 | -0.826 |
|  | *P* value | ＜0.001 | ＜0.001 | 0.030 | ＜0.001 | ＜0.001 | ＜0.001 |
| **Lag –6 days** | | | | | | | |
|  | Spearman correlation coefficient | **0.900** | 0.749 | -0.334 | -0.831 | -0.831 | -0.831 |
|  | *P* value | ＜0.001 | ＜0.001 | 0.013 | ＜0.001 | ＜0.001 | ＜0.001 |
| **Lag –5 days** | | | | | | | |
|  | Spearman correlation coefficient | 0.893 | 0.701 | -0.372 | -0.836 | -0.836 | -0.836 |
|  | *P* value | ＜0.001 | ＜0.001 | 0.005 | ＜0.001 | ＜0.001 | ＜0.001 |
| **Lag –4 days** | | | | | | | |
|  | Spearman correlation coefficient | 0.884 | 0.660 | -0.401 | -0.839 | -0.839 | -0.839 |
|  | *P* value | ＜0.001 | ＜0.001 | 0.002 | ＜0.001 | ＜0.001 | ＜0.001 |
| **Lag –3 days** | | | | | | | |
|  | Spearman correlation coefficient | 0.881 | 0.609 | -0.439 | -0.843 | -0.843 | -0.843 |
|  | *P* value | ＜0.001 | ＜0.001 | 0.001 | ＜0.001 | ＜0.001 | ＜0.001 |
| **Lag –2 days** | | | | | | | |
|  | Spearman correlation coefficient | 0.861 | 0.549 | -0.539 | -0.848 | -0.848 | -0.848 |
|  | *P* value | ＜0.001 | ＜0.001 | ＜0.001 | ＜0.001 | ＜0.001 | ＜0.001 |
| **Lag –1 day** | | | | | | | |
|  | Spearman correlation coefficient | 0.834 | 0.498 | -0.562 | -0.852 | -0.852 | -0.948 |
|  | *P* value | ＜0.001 | ＜0.001 | ＜0.001 | ＜0.001 | ＜0.001 | ＜0.001 |
| **Lag 0** | | | | | | | |
|  | Spearman correlation coefficient | 0.810 | 0.430 | -0.586 | -0.854 | -0.854 | -0.967 |
|  | *P* value | ＜0.001 | 0.001 | ＜0.001 | ＜0.001 | ＜0.001 | ＜0.001 |
| **Lag 1 day** | | | | | | | |
|  | Spearman correlation coefficient | 0.761 | 0.312 | -0.627 | -0.948 | -0.948 | -0.981 |
|  | *P* value | ＜0.001 | 0.015 | ＜0.001 | ＜0.001 | ＜0.001 | ＜0.001 |
| **Lag 2 days** | | | | | | | |
|  | Spearman correlation coefficient | 0.705 | 0.219 | -0.652 | -0.967 | -0.967 | -0.982 |
|  | *P* value | ＜0.001 | 0.095 | ＜0.001 | ＜0.001 | ＜0.001 | ＜0.001 |
| **Lag 3 days** | | | | | | | |
|  | Spearman correlation coefficient | 0.646 | 0.151 | -0.674 | -0.981 | -0.981 | **-0.986** |
|  | *P* value | ＜0.001 | 0.257 | ＜0.001 | ＜0.001 | ＜0.001 | ＜0.001 |
| **Lag 4 days** | | | | | | | |
|  | Spearman correlation coefficient | 0.632 | 0.107 | -0.719 | -0.982 | -0.982 | -0.985 |
|  | *P* value | ＜0.001 | 0.430 | ＜0.001 | ＜0.001 | ＜0.001 | ＜0.001 |
| **Lag 5 days** | | | | | | | |
|  | Spearman correlation coefficient | 0.597 | 0.068 | -0.746 | **-0.986** | **-0.986** | -0.985 |
|  | *P* value | ＜0.001 | 0.618 | ＜0.001 | ＜0.001 | ＜0.001 | ＜0.001 |
| **Lag 6 days** | | | | | | | |
|  | Spearman correlation coefficient | 0.575 | 0.038 | -0.769 | -0.985 | -0.985 | -0.984 |
|  | *P* value | ＜0.001 | 0.783 | ＜0.001 | ＜0.001 | ＜0.001 | ＜0.001 |
| **Lag 7 days** | | | | | | | |
|  | Spearman correlation coefficient | 0.545 | -0.003 | -0.792 | -0.985 | -0.985 | -0.983 |
|  | *P* value | ＜0.001 | 0.982 | ＜0.001 | ＜0.001 | ＜0.001 | ＜0.001 |
| **Lag 8 days** | | | | | | | |
|  | Spearman correlation coefficient | 0.520 | -0.041 | -0.813 | -0.984 | -0.984 | -0.982 |
|  | *P* value | ＜0.001 | 0.770 | ＜0.001 | ＜0.001 | ＜0.001 | ＜0.001 |
| **Lag 9 days** | | | | | | | |
|  | Spearman correlation coefficient | 0.495 | -0.099 | -0.837 | -0.983 | -0.983 | -0.981 |
|  | *P* value | ＜0.001 | 0.485 | ＜0.001 | ＜0.001 | ＜0.001 | ＜0.001 |
| **Lag 10 days** | | | | | | | |
|  | Spearman correlation coefficient | 0.466 | -0.134 | -0.859 | -0.982 | -0.982 | -0.980 |
|  | *P* value | 0.001 | 0.349 | ＜0.001 | ＜0.001 | ＜0.001 | ＜0.001 |
| **Lag 11 days** | | | | | | | |
|  | Spearman correlation coefficient | 0.441 | -0.18 | -0.880 | -0.981 | -0.981 | -0.979 |
|  | *P* value | 0.001 | 0.210 | ＜0.001 | ＜0.001 | ＜0.001 | ＜0.001 |
| **Lag 12 days** | | | | | | | |
|  | Spearman correlation coefficient | 0.411 | -0.24 | -0.898 | -0.980 | -0.980 | -0.978 |
|  | *P* value | 0.003 | 0.097 | ＜0.001 | ＜0.001 | ＜0.001 | ＜0.001 |
| **Lag 13 days** | | | | | | | |
|  | Spearman correlation coefficient | 0.373 | -0.303 | -0.914 | -0.979 | -0.979 | -0.978 |
|  | *P* value | 0.009 | 0.037 | ＜0.001 | ＜0.001 | ＜0.001 | ＜0.001 |
| **Lag 14 days** | | | | | | | |
|  | Spearman correlation coefficient | 0.315 | -0.341 | -0.924 | -0.978 | -0.978 | -0.977 |
|  | *P* value | 0.031 | 0.019 | ＜0.001 | ＜0.001 | ＜0.001 | ＜0.001 |
| **Lag 15 days** | | | | | | | |
|  | Spearman correlation coefficient | 0.279 | -0.377 | -0.953 | -0.978 | -0.978 | -0.978 |
|  | *P* value | 0.060 | 0.010 | ＜0.001 | ＜0.001 | ＜0.001 | ＜0.001 |
| **Lag 16 days** | | | | | | | |
|  | Spearman correlation coefficient | 0.257 | -0.438 | **-0.973** | -0.977 | -0.977 | -0.976 |
|  | *P* value | 0.088 | 0.003 | ＜0.001 | ＜0.001 | ＜0.001 | ＜0.001 |
| **Lag 17 days** | | | | | | | |
|  | Spearman correlation coefficient | 0.206 | -0.471 | -0.965 | -0.978 | -0.978 | -0.974 |
|  | *P* value | 0.181 | 0.001 | ＜0.001 | ＜0.001 | ＜0.001 | ＜0.001 |
| **Lag 18 days** | | | | | | | |
|  | Spearman correlation coefficient | 0.156 | -0.524 | -0.964 | -0.976 | -0.976 | -0.972 |
|  | *P* value | 0.318 | ＜0.001 | ＜0.001 | ＜0.001 | ＜0.001 | ＜0.001 |
| **Lag 19 days** | | | | | | | |
|  | Spearman correlation coefficient | 0.086 | -0.565 | -0.971 | -0.974 | -0.974 | -0.971 |
|  | *P* value | 0.59 | ＜0.001 | ＜0.001 | ＜0.001 | ＜0.001 | ＜0.001 |
| **Lag 20 days** | | | | | | | |
|  | Spearman correlation coefficient | 0.049 | -0.633 | -0.963 | -0.972 | -0.972 | -0.969 |
|  | *P* value | 0.763 | ＜0.001 | ＜0.001 | ＜0.001 | ＜0.001 | ＜0.001 |

Table 2 The time lag cross-correlation between the SMI and COVID-19-related data from 20 January to 20 March in 2020.

| Sina Micro Index | | New confirmed cases | New death cases | New cured discharge cases | Cumulative confirmed cases | Cumulative death cases | Cumulative cured discharge cases |
| --- | --- | --- | --- | --- | --- | --- | --- |
| **Lag –20 days** | | | | | | | |
|  | Spearman correlation coefficient | 0.741 | 0.736 | 0.259 | -0.693 | -0.693 | -0.693 |
|  | *P* value | ＜0.001 | ＜0.001 | 0.102 | ＜0.001 | ＜0.001 | ＜0.001 |
| **Lag –19 days** | | | | | | | |
|  | Spearman correlation coefficient | **0.753** | **0.739** | 0.136 | -0.690 | -0.690 | -0.690 |
|  | *P* value | ＜0.001 | ＜0.001 | 0.392 | ＜0.001 | ＜0.001 | ＜0.001 |
| **Lag –18 days** | | | | | | | |
|  | Spearman correlation coefficient | 0.728 | 0.738 | -0.005 | -0.666 | -0.666 | -0.666 |
|  | *P* value | ＜0.001 | ＜0.001 | 0.974 | ＜0.001 | ＜0.001 | ＜0.001 |
| **Lag –17 days** | | | | | | | |
|  | Spearman correlation coefficient | 0.697 | 0.735 | -0.065 | -0.671 | -0.671 | -0.671 |
|  | *P* value | ＜0.001 | ＜0.001 | 0.677 | ＜0.001 | ＜0.001 | ＜0.001 |
| **Lag –16 days** | | | | | | | |
|  | Spearman correlation coefficient | 0.690 | 0.728 | -0.122 | -0.678 | -0.678 | -0.678 |
|  | *P* value | ＜0.001 | ＜0.001 | 0.424 | ＜0.001 | ＜0.001 | ＜0.001 |
| **Lag –15 days** | | | | | | | |
|  | Spearman correlation coefficient | 0.638 | 0.681 | -0.212 | -0.672 | -0.672 | -0.672 |
|  | *P* value | ＜0.001 | ＜0.001 | 0.158 | ＜0.001 | ＜0.001 | ＜0.001 |
| **Lag –14 days** | | | | | | | |
|  | Spearman correlation coefficient | 0.640 | 0.669 | -0.259 | -0.681 | -0.681 | -0.681 |
|  | *P* value | ＜0.001 | ＜0.001 | 0.079 | ＜0.001 | ＜0.001 | ＜0.001 |
| **Lag –13 days** | | | | | | | |
|  | Spearman correlation coefficient | 0.658 | 0.684 | -0.307 | -0.696 | -0.696 | -0.696 |
|  | *P* value | ＜0.001 | ＜0.001 | 0.034 | ＜0.001 | ＜0.001 | ＜0.001 |
| **Lag –12 days** | | | | | | | |
|  | Spearman correlation coefficient | 0.668 | 0.648 | -0.356 | -0.703 | -0.703 | -0.703 |
|  | *P* value | ＜0.001 | ＜0.001 | 0.012 | ＜0.001 | ＜0.001 | ＜0.001 |
| **Lag –11 days** | | | | | | | |
|  | Spearman correlation coefficient | 0.684 | 0.635 | -0.397 | **-0.707** | **-0.707** | **-0.707** |
|  | *P* value | ＜0.001 | ＜0.001 | 0.004 | ＜0.001 | ＜0.001 | ＜0.001 |
| **Lag –10 days** | | | | | | | |
|  | Spearman correlation coefficient | 0.685 | 0.552 | -0.492 | -0.667 | -0.667 | -0.667 |
|  | *P* value | ＜0.001 | ＜0.001 | ＜0.001 | ＜0.001 | ＜0.001 | ＜0.001 |
| **Lag –9 days** | | | | | | | |
|  | Spearman correlation coefficient | 0.689 | 0.511 | -0.545 | -0.666 | -0.666 | -0.666 |
|  | *P* value | ＜0.001 | ＜0.001 | ＜0.001 | ＜0.001 | ＜0.001 | ＜0.001 |
| **Lag –8 days** | | | | | | | |
|  | Spearman correlation coefficient | 0.709 | 0.503 | -0.580 | -0.654 | -0.654 | -0.654 |
|  | *P* value | ＜0.001 | ＜0.001 | ＜0.001 | ＜0.001 | ＜0.001 | ＜0.001 |
| **Lag –7 days** | | | | | | | |
|  | Spearman correlation coefficient | 0.675 | 0.447 | -0.615 | -0.653 | -0.653 | -0.653 |
|  | *P* value | ＜0.001 | 0.001 | ＜0.001 | ＜0.001 | ＜0.001 | ＜0.001 |
| **Lag –6 days** | | | | | | | |
|  | Spearman correlation coefficient | 0.684 | 0.389 | -0.627 | -0.645 | -0.645 | -0.645 |
|  | *P* value | ＜0.001 | 0.003 | ＜0.001 | ＜0.001 | ＜0.001 | ＜0.001 |
| **Lag –5 days** | | | | | | | |
|  | Spearman correlation coefficient | 0.645 | 0.333 | -0.629 | -0.644 | -0.644 | -0.644 |
|  | *P* value | ＜0.001 | 0.012 | ＜0.001 | ＜0.001 | ＜0.001 | ＜0.001 |
| **Lag –4 days** | | | | | | | |
|  | Spearman correlation coefficient | 0.616 | 0.238 | -0.618 | -0.647 | -0.647 | -0.647 |
|  | *P* value | ＜0.001 | 0.075 | ＜0.001 | ＜0.001 | ＜0.001 | ＜0.001 |
| **Lag –3 days** | | | | | | | |
|  | Spearman correlation coefficient | 0.607 | 0.177 | -0.636 | -0.636 | -0.636 | -0.636 |
|  | *P* value | ＜0.001 | 0.183 | ＜0.001 | ＜0.001 | ＜0.001 | ＜0.001 |
| **Lag –2 days** | | | | | | | |
|  | Spearman correlation coefficient | 0.571 | 0.081 | **-0.722** | -0.626 | -0.626 | -0.626 |
|  | *P* value | ＜0.001 | 0.539 | ＜0.001 | ＜0.001 | ＜0.001 | ＜0.001 |
| **Lag –1 day** | | | | | | | |
|  | Spearman correlation coefficient | 0.481 | -0.003 | -0.719 | -0.614 | -0.614 | -0.697 |
|  | *P* value | ＜0.001 | 0.982 | ＜0.001 | ＜0.001 | ＜0.001 | ＜0.001 |
| **Lag 0** | | | | | | | |
|  | Spearman correlation coefficient | 0.446 | -0.113 | -0.702 | -0.605 | -0.605 | -0.683 |
|  | *P* value | ＜0.001 | 0.385 | ＜0.001 | ＜0.001 | ＜0.001 | ＜0.001 |
| **Lag 1 day** | | | | | | | |
|  | Spearman correlation coefficient | 0.352 | -0.256 | -0.717 | -0.687 | -0.687 | -0.668 |
|  | *P* value | 0.006 | 0.049 | ＜0.001 | ＜0.001 | ＜0.001 | ＜0.001 |
| **Lag 2 days** | | | | | | | |
|  | Spearman correlation coefficient | 0.284 | -0.315 | -0.703 | -0.683 | -0.683 | -0.650 |
|  | *P* value | 0.029 | 0.015 | ＜0.001 | ＜0.001 | ＜0.001 | ＜0.001 |
| **Lag 3 days** | | | | | | | |
|  | Spearman correlation coefficient | 0.226 | -0.335 | -0.695 | -0.668 | -0.668 | -0.632 |
|  | *P* value | 0.087 | 0.010 | ＜0.001 | ＜0.001 | ＜0.001 | ＜0.001 |
| **Lag 4 days** | | | | | | | |
|  | Spearman correlation coefficient | 0.177 | -0.362 | -0.665 | -0.650 | -0.650 | -0.612 |
|  | *P* value | 0.187 | 0.006 | ＜0.001 | ＜0.001 | ＜0.001 | ＜0.001 |
| **Lag 5 days** | | | | | | | |
|  | Spearman correlation coefficient | 0.133 | -0.427 | -0.653 | -0.632 | -0.632 | -0.590 |
|  | *P* value | 0.328 | 0.001 | ＜0.001 | ＜0.001 | ＜0.001 | ＜0.001 |
| **Lag 6 days** | | | | | | | |
|  | Spearman correlation coefficient | 0.038 | -0.406 | -0.620 | -0.612 | -0.612 | -0.568 |
|  | *P* value | 0.786 | 0.002 | ＜0.001 | ＜0.001 | ＜0.001 | ＜0.001 |
| **Lag 7 days** | | | | | | | |
|  | Spearman correlation coefficient | -0.051 | -0.435 | -0.562 | -0.590 | -0.590 | -0.543 |
|  | *P* value | 0.712 | 0.001 | ＜0.001 | ＜0.001 | ＜0.001 | ＜0.001 |
| **Lag 8 days** | | | | | | | |
|  | Spearman correlation coefficient | -0.111 | -0.447 | -0.548 | -0.568 | -0.568 | -0.517 |
|  | *P* value | 0.431 | 0.001 | ＜0.001 | ＜0.001 | ＜0.001 | ＜0.001 |
| **Lag 9 days** | | | | | | | |
|  | Spearman correlation coefficient | -0.125 | -0.490 | -0.496 | -0.543 | -0.543 | -0.489 |
|  | *P* value | 0.376 | ＜0.001 | ＜0.001 | ＜0.001 | ＜0.001 | ＜0.001 |
| **Lag 10 days** | | | | | | | |
|  | Spearman correlation coefficient | -0.182 | -0.537 | -0.472 | -0.517 | -0.517 | -0.459 |
|  | *P* value | 0.201 | ＜0.001 | 0.001 | ＜0.001 | ＜0.001 | 0.001 |
| **Lag 11 days** | | | | | | | |
|  | Spearman correlation coefficient | -0.222 | -0.485 | -0.436 | -0.489 | -0.489 | -0.426 |
|  | *P* value | 0.122 | ＜0.001 | 0.002 | ＜0.001 | ＜0.001 | 0.003 |
| **Lag 12 days** | | | | | | | |
|  | Spearman correlation coefficient | -0.282 | -0.538 | -0.405 | -0.459 | -0.459 | -0.390 |
|  | *P* value | 0.049 | ＜0.001 | 0.005 | 0.001 | 0.001 | 0.007 |
| **Lag 13 days** | | | | | | | |
|  | Spearman correlation coefficient | -0.248 | -0.565 | -0.343 | -0.426 | -0.426 | -0.356 |
|  | *P* value | 0.089 | ＜0.001 | 0.021 | 0.003 | 0.003 | 0.015 |
| **Lag 14 days** | | | | | | | |
|  | Spearman correlation coefficient | -0.257 | -0.504 | -0.315 | -0.390 | -0.390 | -0.312 |
|  | *P* value | 0.081 | ＜0.001 | 0.037 | 0.007 | 0.007 | 0.037 |
| **Lag 15 days** | | | | | | | |
|  | Spearman correlation coefficient | -0.342 | -0.515 | -0.249 | -0.356 | -0.356 | -0.270 |
|  | *P* value | 0.020 | ＜0.001 | 0.108 | 0.015 | 0.015 | 0.076 |
| **Lag 16 days** | | | | | | | |
|  | Spearman correlation coefficient | -0.401 | -0.389 | -0.193 | -0.312 | -0.312 | -0.218 |
|  | *P* value | 0.006 | 0.008 | 0.220 | 0.037 | 0.037 | 0.160 |
| **Lag 17 days** | | | | | | | |
|  | Spearman correlation coefficient | -0.326 | -0.417 | -0.121 | -0.270 | -0.270 | -0.161 |
|  | *P* value | 0.031 | 0.005 | 0.451 | 0.076 | 0.076 | 0.308 |
| **Lag 18 days** | | | | | | | |
|  | Spearman correlation coefficient | -0.386 | -0.332 | -0.074 | -0.218 | -0.218 | -0.111 |
|  | *P* value | 0.011 | 0.030 | 0.649 | 0.160 | 0.160 | 0.488 |
| **Lag 19 days** | | | | | | | |
|  | Spearman correlation coefficient | -0.338 | -0.246 | 0.023 | -0.161 | -0.161 | -0.059 |
|  | *P* value | 0.029 | 0.117 | 0.891 | 0.308 | 0.308 | 0.715 |
| **Lag 20 days** | | | | | | | |
|  | Spearman correlation coefficient | -0.442 | -0.245 | 0.088 | -0.111 | -0.111 | 0.015 |
|  | *P* value | 0.004 | 0.123 | 0.601 | 0.488 | 0.488 | 0.929 |
