# Supplementary material for: Application of the Internet Platform in Monitoring Chinese Public Attention to the Outbreak of COVID-19
Source: Front Public Health. 2022 Jan 28;9:755530. doi: 10.3389/fpubh.2021.755530 (PMC8831856; doi:10.3389/fpubh.2021.755530)
Supplement: Supplementary file 2 [file Data_Sheet_2.DOCX]

Appendix 2

Table 1 Forecast results of four multiple linear regression models.

| Date | New confirmed cases | Model 1  predicted values | Model 3  predicted values | New death cases | Model 2  predicted values | Model 4  predicted values |
| --- | --- | --- | --- | --- | --- | --- |
| 20-Jan | 77 | 166 | 844 | 2 | -37 | 42 |
| 21-Jan | 149 | 275 | 1113 | 3 | -34 | 8 |
| 22-Jan | 131 | 1758 | 155 | 8 | -28 | -19 |
| 23-Jan | 259 | -1302 | 2248 | 8 | -13 | 26 |
| 24-Jan | 444 | 252 | 2272 | 16 | -1 | 44 |
| 25-Jan | 688 | 430 | 2624 | 15 | 30 | 52 |
| 26-Jan | 769 | 1174 | 1756 | 24 | 50 | 25 |
| 27-Jan | 1771 | 1080 | 1452 | 26 | 54 | 21 |
| 28-Jan | 1459 | 2405 | 1154 | 26 | 74 | 11 |
| 29-Jan | 1737 | 2198 | 952 | 38 | 77 | 15 |
| 30-Jan | 1982 | 1997 | 1622 | 43 | 78 | 39 |
| 31-Jan | 2102 | 2302 | 2578 | 46 | 86 | 68 |
| 1-Feb | 2590 | 3806 | 3932 | 45 | 82 | 72 |
| 2-Feb | 2829 | 4076 | 2686 | 57 | 81 | 61 |
| 3-Feb | 3235 | 4050 | 3591 | 64 | 80 | 93 |
| 4-Feb | 3887 | 3536 | 2536 | 65 | 78 | 71 |
| 5-Feb | 3694 | 3733 | 3345 | 73 | 80 | 94 |
| 6-Feb | 3143 | 3735 | 1529 | 73 | 82 | 28 |
| 7-Feb | 3399 | 3746 | 1592 | 86 | 84 | 17 |
| 8-Feb | 2656 | 3830 | 3190 | 89 | 79 | 105 |
| 9-Feb | 3062 | 3333 | 3593 | 97 | 77 | 117 |
| 10-Feb | 2478 | 3332 | 2595 | 108 | 77 | 90 |
| 11-Feb | 2015 | 3282 | 2984 | 97 | 75 | 118 |
| 12-Feb | 15152 | 2592 | 2640 | 254 | 72 | 111 |
| 13-Feb | 5090 | 5552 | 3523 | 121 | 122 | 137 |
| 14-Feb | 2641 | 3049 | 2060 | 143 | 87 | 92 |
| 15-Feb | 2009 | 1447 | 2765 | 142 | 66 | 110 |
| 16-Feb | 2048 | 1162 | 2366 | 105 | 63 | 99 |
| 17-Feb | 1886 | 1173 | 2106 | 98 | 64 | 84 |
| 18-Feb | 1749 | 694 | 1477 | 136 | 56 | 85 |
| 19-Feb | 394 | 602 | 2102 | 114 | 54 | 94 |
| 20-Feb | 889 | 1348 | 1636 | 118 | 67 | 78 |
| 21-Feb | 397 | 1619 | 1929 | 109 | 71 | 100 |
| 22-Feb | 648 | 769 | 1518 | 97 | 57 | 71 |
| 23-Feb | 409 | 645 | 1151 | 150 | 54 | 59 |
| 24-Feb | 508 | 1264 | 553 | 71 | 67 | 60 |
| 25-Feb | 406 | 578 | 1345 | 52 | 55 | 63 |
| 26-Feb | 433 | 715 | -584 | 29 | 58 | 37 |
| 27-Feb | 327 | 586 | 621 | 44 | 56 | 54 |
| 28-Feb | 427 | 830 | 91 | 47 | 59 | 36 |
| 29-Feb | 573 | 564 | -1928 | 35 | 54 | -20 |
| 1-Mar | 202 | 679 | 297 | 42 | 56 | 60 |
| 2-Mar | 125 | 905 | -194 | 31 | 60 | 52 |
| 3-Mar | 119 | 663 | -584 | 38 | 55 | 35 |
| 4-Mar | 139 | 892 | 121 | 31 | 60 | 5 |
| 5-Mar | 143 | 418 | -86 | 30 | 51 | 24 |
| 6-Mar | 99 | 331 | 924 | 28 | 50 | 67 |
| 7-Mar | 44 | -237 | 919 | 27 | 40 | 78 |
| 8-Mar | 40 | -361 | 1460 | 22 | 38 | 87 |
| 9-Mar | 19 | 235 | 1140 | 17 | 48 | 49 |
| 10-Mar | 24 | 42 | 986 | 22 | 46 | 82 |
| 11-Mar | 15 | -95 | -76 | 11 | 43 | 40 |
| 12-Mar | 8 | -22 | -1029 | 7 | 44 | 3 |
| 13-Mar | 11 | 175 | -584 | 13 | 47 | 3 |
| 14-Mar | 20 | -277 | -432 | 10 | 39 | 9 |
| 15-Mar | 16 | -262 | -670 | 14 | 39 | 4 |
| 16-Mar | 21 | 244 | -593 | 13 | 48 | 27 |
| 17-Mar | 13 | 7 | 1101 | 11 | 44 | 51 |
| 18-Mar | 34 | -67 | 637 | 8 | 43 | 42 |
| 19-Mar | 39 | -29 | 1768 | 3 | 44 | 64 |
| 20-Mar | 41 | 285 | 634 | 7 | 50 | 45 |
